# A qualitative systematic review of studies using the normalization process theory to research implementation processes

**DOI:** 10.1186/1748-5908-9-2

**Published:** 2014-01-02

**Authors:** Rachel McEvoy, Luciana Ballini, Susanna Maltoni, Catherine A O’Donnell, Frances S Mair, Anne MacFarlane

**Affiliations:** 1 Graduate Entry Medical School, 4i Research Center, University of Limerick, Limerick, Ireland; 2 Responsabile di Area, Osservatorio Regionale per l’Innovazione (ORI), Agenzia sanitaria e sociale regionale, viale Aldo Moro 21-40127, Bologna, Italy; 3 General Practice and Primary Care, Institute of Health and Wellbeing, MVLS. University of Glasgow, 1 Horselethill Road, G12 9LX, Glasgow, Scotland

**Keywords:** Implementation, Policy, Normalization process theory, Theory, Translational gaps

## Abstract

**Background:**

There is a well-recognized need for greater use of theory to address research translational gaps. Normalization Process Theory (NPT) provides a set of sociological tools to understand and explain the social processes through which new or modified practices of thinking, enacting, and organizing work are implemented, embedded, and integrated in healthcare and other organizational settings. This review of NPT offers readers the opportunity to observe how, and in what areas, a particular theoretical approach to implementation is being used. In this article we review the literature on NPT in order to understand what interventions NPT is being used to analyze, how NPT is being operationalized, and the reported benefits, if any, of using NPT.

**Methods:**

Using a framework analysis approach, we conducted a qualitative systematic review of peer-reviewed literature using NPT. We searched 12 electronic databases and all citations linked to six key NPT development papers. Grey literature/unpublished studies were not sought. Limitations of English language, healthcare setting and year of publication 2006 to June 2012 were set.

**Results:**

Twenty-nine articles met the inclusion criteria; in the main, NPT is being applied to qualitatively analyze a diverse range of complex interventions, many beyond its original field of e-health and telehealth. The NPT constructs have high stability across settings and, notwithstanding challenges in applying NPT in terms of managing overlaps between constructs, there is evidence that it is a beneficial heuristic device to explain and guide implementation processes.

**Conclusions:**

NPT offers a generalizable framework that can be applied across contexts with opportunities for incremental knowledge gain over time and an explicit framework for analysis, which can explain and potentially shape implementation processes. This is the first review of NPT in use and it generates an impetus for further and extended use of NPT. We recommend that in future NPT research, authors should explicate their rationale for choosing NPT as their theoretical framework and, where possible, involve multiple stakeholders including service users to enable analysis of implementation from a range of perspectives.

## Background

There has been a proliferation of research about research-practice-policy links in recent decades [1]. In spite of the growth in literature, there remains a well-recognized and significant translational gap between these domains. This gap has captured the attention of policy makers and researchers alike, with repeated calls for the greater use of explicit theory in research that explores implementation processes [2-4]. The proposed benefits are that theory can offer us generalizable frameworks that can apply across differing settings and individuals; the opportunity for incremental accumulation of knowledge; and an explicit framework for analysis [2,5]. Also, using theory may enhance our understanding of barriers to implementation, but more than that, it may enhance our ability to design interventions and explore mediating pathways to shape and improve implementation processes [6,7]. It is thus important both to develop and test new theories that are in use, to appraise their relevance and utility for the field of implementation research [8-10].

One such new theory presented in the literature is the Normalization Process Theory (NPT). NPT is a sociological theory that has been widely promoted as a means to understand implementation, embedding and integration of innovation in healthcare settings, and has been advocated as a means of bridging the translational gap [8,10]. It has potential utility as a conceptual framework to explore the gap between health research evidence, policy, and practice because epistemologically, it emphasizes the fluid, dynamic and interactive processes between context, actors and objects that is congruent with interactive and social models of research use [11]; it is derived from studies seeking to understand the implementation of innovation and complex interventions in healthcare settings so it is highly attuned to the specifics of this organizational setting; and it encourages the recommended whole-system perspective on implementation research [3]. In the next section, we provide an overview of NPT before going on to describe our research objectives and the methods of our review.

### From Normalization Process Model (NPM) to Normalization Process Theory (NPT)

The Normalization Process Model (NPM) was initially developed as an applied theoretical model to assist clinicians and researchers to understand and evaluate the factors that inhibit and promote the routine incorporation of complex healthcare interventions in practice [12-14]. Much of the early work was related to implementation of e-health applications.

The further empirical applications of the NPM showed that while it could explain factors that promote and inhibit ‘collective action’ (*i.e.*, the distribution of work required among stakeholders and the resources to support that), it did not address how participants understood and came to engage and support a new practice and how they reflected on and evaluated it. Through the development of further constructs (see NPT theoretical constructs from Finch, Mair, *et al*. [15]), accounting for how people understand and make sense of a practice (*i.e.*, Coherence), engage and participate with it (*i.e.*, Cognitive Participation), and reflect or appraise its effects (*i.e.*, Reflexive Monitoring), the model became a theory, *i.e.*, NPT. For the most part, the term NPT is used through out this paper, unless otherwise stated.

### NPT theoretical constructs (from Finch, Mair *et al.*)

1. **Coherence**: the process and work of sense-making and understanding that individuals and organisations have to go through in order to promote or inhibit the routine embedding of a practice.

2. **Cognitive Participation**: the process and work that individuals and organisations have to go through in order to enrol individuals to engage with the new practice.

3. **Collective Action**: the work that individuals and organisations have to do to enact the new practice. (“Collective Action” was initially referred to as NPM, and consisted of four subcomponents (i.e. Contextual Integration (CI), Relational Integration (RI), Interactional Workability (IW), and Skill Set Workability (SSW)). For a more detailed description of NPM see May [12]).

4. **Reflexive Monitoring**: the work inherent in the informal and formal appraisal of a new practice once it is in use, in order to assess its advantages and disadvantages, and which develops users’ comprehension of the effects of a practice.

Since then, the theory’s development has focused on building a middle-range theory that explains how material practices (the things that people do when they implement complex healthcare interventions) become routinely embedded in their social contexts as the result of people working, individually and collectively, to enact them [13,14].

**Figure 1:**
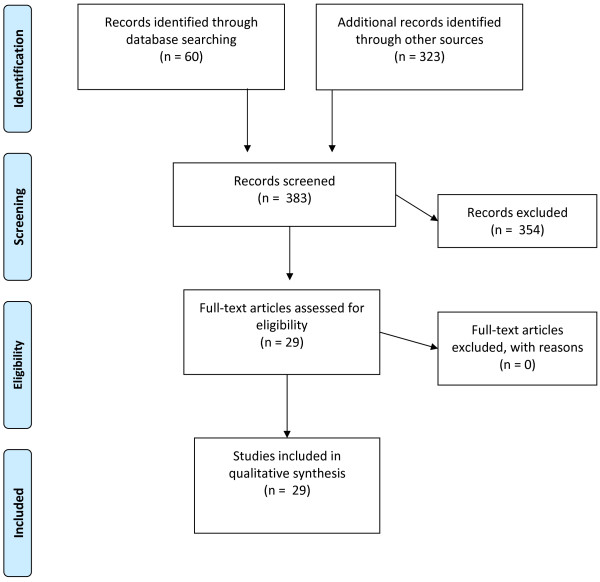
PRISMA flow diagram of study selection utilizing NPT/NPM.

Given its sociological origins, NPT is not focused on the relationship between individual attitudes and intentions and behavioral outcomes, which is the concern of psychological theories such as the Theory of Planned Behavior [16]. Like the sociological theory of Communities of Practice [17], NPT does pay attention to how knowledge is held, transferred, and created within and across professional groups, but it also seeks to understand the work that actors (clinicians, implementers, and patients alike) have to engage in to implement new knowledge in practice [18,19]. Similar to theories of actor networks and diffusion of innovation [20,21], NPT pays attention to the legitimacy of the intervention and the role of opinion leaders; it is concerned with understanding trust and interpersonal relationships within social networks as they impact on the introduction of innovation [22,23]. However, NPT extends beyond the initial introduction of innovation to investigate the processes by which innovation may become embedded and routinized in practice, so much so that it becomes regarded as a normal and taken-for-granted way of working. Among NPT’s distinctive features is the attention to all stakeholders’ involvement in implementation processes, the work that they have to do individually and collectively, and the subtle and gradual processes from embedding and integrating to normalization [13]. The theory is centered on understanding social phenomena defined by four theoretical constructs, which characterize mechanisms that are energized by investments made by individuals and organizations (see Figure 1).

The development of NPT [14] focused on addressing two key criteria for theory to be ‘useful’: that it must offer adequate description and be fit for purpose. Thus, as outlined by Finch, Mair *et al.*[15], the theory has been developed to offer transparent and transferable explanations for the phenomena of interest (processes of embedding new practice and ways of working) revealed by empirical investigation.

There is a growing community of researchers who have made an *a priori* choice to use NPT in their research. Given the aforementioned calls to investigate new theories, we consider it timely to review how this particular new theory is being operationalized and, importantly, explore what benefits, if any, are seen to derive from its utilization. This review of NPT contributes to a body of knowledge about how theory may benefit implementation research, and if so what should the research agenda now be for NPT and other relevant theories in the field. Therefore, in this article our research objectives are to review the literature on NPT (see Additional file 1: PICO Table) in order to understand what interventions NPT is being used to analyze, how NPT is being operationalized and the reported benefits, if any, of using NPT.

## Methods

We conducted a qualitative descriptive review of peer-reviewed NPT literature in the English language published from 2006 up to June 1st 2012.

### Search terms and strategy

Utilizing the interface engine EBSCOhost, the first author (RM) completed an advanced search incorporating the following electronic bibliographic databases: Academic Search Complete; AMED—The Allied and Complementary Medicine Database; Biomedical Reference Collection: Expanded; CINAHL Plus with Full Text; MEDLINE; OmniFile Full Text Mega (H.W.Wilson); PsycARTICLES; PsycINFO; Social Sciences Full Text (H.W. Wilson); UK and Ireland Reference Center (see Additional file 2). A search of both Embase and Pubmed was also carried out. In the search process, the terms ‘Normalization Process Theory’ or ‘Normalization Process Model’ were used. The first author (RM) also screened all citations linked to six key concept papers that were specific to the development of the theory/model [10,12-14,24,25]. Citations were also solicited from academics involved in the development of NPT. Google Scholar alerts were further activated during the search process, which commenced in February 2012 and ended on June 1st 2012. The final list of articles was then circulated to a number of experts in the field of NPT for review and no additional papers were noted. The issue of discrepancies did not arise.

### Inclusion and exclusion criteria

Papers on the subject of ‘Normalization Process Theory’ or ‘Normalization Process Model’ in a health-related field that met all the following criteria were included: Published peer-reviewed empirical papers or papers that may be in press or accepted for publication; and an explicit reference to NPT or NPM in the article heading, abstract or keywords.

Given the decision to review the application of NPT in completed empirical work, study protocols, editorials, discussion/briefing papers, conference papers, or core concept papers that describe the theory and its development were not included (*e.g.*, [13]).

### Data abstraction and framework analysis

Citations were downloaded into EndNote reference manager software, and full documents were imported into NVivo 10 software for analysis.

Given our interest in identifying *a priori* themes (*i.e.*, the four NPT constructs), we adopted a framework analysis approach [26]. We also sought out emergent issues (*e.g.*, country of origin, research focus, stakeholder involvement) that were considered relevant to the objectives of our review.

This framework analysis approach incorporated the following five key stages: familiarization, identifying a thematic framework, indexing, charting, and mapping and interpretation. For indexing and charting, the first author extracted material from any part of the paper that was relevant to an *a priori* or emergent theme. For the *a priori* themes, only data that were explicitly connected to NPT by the authors of the identified papers were coded.

The final stage of mapping and interpretation focused primarily on our *a priori* themes and had two objectives. First, to examine stability of the constructs across studies, we explored how NPT was being operationalized across settings. For this we analyzed whether data coded explicitly by authors as a particular construct had resonance with our understanding of that construct as per the core NPT papers. We also compared authors’ accounts of each construct as applied to their study setting and any relevant corresponding data. Given the subjectivity involved in interpretive analysis processes, our aim was to see if we could understand authors’ coding decisions rather than judging them to be ‘correct’ or ‘incorrect’ *per se.* Second, to determine whether there were any benefits to using NPT we focused on the authors’ reflections, usually located in the discussion section of the papers.

The mapping and interpretation of the data, and recommendations, were discussed with all co-authors and refined until consensus was reached.

## Results

### Search results

From the 383 records screened, 354 were excluded and 29 full-text articles that met the inclusion criteria were retrieved (see Figure 1) (see Additional file 1: PICO Table). Due to the qualitative nature of the literature being reviewed, PICO as a search strategy did not neatly fit with our research questions, so we adapted the PICO Table to include the following criteria: participants, study design and collection approach, interventions, analysis, aims/discussion, and outcomes.

Drawing on Hawker, Payne, *et al.*[27] (see Additional file 3: Quality Appraisal Tool), each of the 29 papers was subjected to a quality appraisal process undertaken by three of the authors (RM, AMacF and LB). This process enabled us to make an informed decision about the quality of the reported research. All 29 papers were included, with scores ranging from 18 to 36 (maximum score being 36) and a mean score of 29.06. Study-specific appraisal results are included in Additional file 3. Overall, these were good quality studies.

To ensure consistency during the data abstraction and framework analysis phase of work, one-fifth of the articles were double-coded independently. Coding agreement was close to 100%.

### Findings as per the research objectives

#### What interventions is NPT being used to analyze?

Of the 29 articles appraised, 21 originated in the UK (the country of origin for NPT), five in Australia, and one each in Ireland, South Africa, and The Netherlands (see Table 1). NPT is mainly being used in qualitative research to study the implementation of complex interventions that introduce a new way of working in healthcare settings (see Table 1). This includes eight studies [15,18,28-33] in the field of e-health and telehealthcare and the remaining 21 studies [5,19,34-52] in several other healthcare fields, for example chronic health care, maternity care, and language interpretation services. Three of the papers reported the use of NPT to inform the development of tools that support implementation work [15,31,33].

**Table 1:** Author, country of origin, topic and research focus of papers included in the review

**Author**	**Country of origin**	**Topic**	**Research focus**
Mair *et al.*[28]	UK	E-health	A systematic review of reviews of e-health implementation studies, focusing on implementation processes rather than outcomes.
Blakeman *et al.*[34]	UK	Chronic kidney disease in primary care	Qualitative interview study in general practices participating in a chronic kidney disease (CKD) collaborative, that aims to explore processes underpinning the implementation of CKD management in primary care.
Franx *et al.*[35]	The Netherlands	Primary care: stepped-care treatment	An intervention study using a controlled before and after design. Part of the study was a process evaluation utilizing semi-structured group interviews to provide insight into the perceptions of the participating clinicians of the implementation of stepped cared for depression into their daily routines.
Ehrlich *et al.*[36]	Australia	Registered nurses in general practice	A qualitative focus group study designed to develop understanding about how a registered nurse-provided care coordination model can ‘fit’ within organizational processes and professional relationships in general practice.
Finch *et al.*[15]	UK	E-health	This paper describes the process and outcome of a project to develop a theory-based instrument for measuring implementation processes relating to e-health interventions, and identifies key issues and methodological challenges for advancing work in this field. A 30-item instrument (Technology Adoption Readiness Scale (TARS)) for measuring normalization processes in the context of e-health service interventions was developed and pre-tested in two professional samples.
Gallacher *et al.*[19]	UK	Chronic heart failure	A secondary analysis of qualitative interview data to assess the burden associated with treatment among patients living with chronic heart failure.
Watson *et al.*[37]	UK	Transitional care for young people	Scoping review of the evidence to identify successful models of transitional care for young people with complex healthcare needs. Three conditions were used as exemplars: cerebral palsy, autism spectrum disorders, and diabetes.
Forster *et al.*[5]	Australia	Maternity care	Authors use two case studies where new models of maternity care were implemented and evaluated via randomized controlled trials (RCTs) to discuss how (or whether) the use of theory might inform implementation and sustainability strategies.
Atkins *et al.*[38]	South Africa	TB treatment	A qualitative interview and focus group study documenting providers’ experiences of the implementation of a new tuberculosis treatment programme.
Godden and King [29]	UK	Telehealth in respiratory medicine	To determine the potential for applying telehealth in a region of the UK by exploring the distribution of patients and examining attitudes to implementation of telehealth.
James [39]	UK	Speech and language therapy	A review and data synthesis of qualitative research data on a speech and language intervention.
MacFarlane and O’Reilly-de Brún [40]	Ireland	Language interpretation services	A reflexive account of the authors’ experience of using a theory-driven conceptual framework, in a qualitative evaluation of general practitioners’ uptake of a free pilot language interpreting service. Authors conducted an inductive thematic analysis using the constant comparative method.
Murray *et al.*[18]	UK	E-health initiatives	A qualitative semi-structured interview study, using a case study methodology. Three case studies were selected to provide a range of healthcare contexts to assess factors that promote or inhibit the successful implementation, embedding, and integration of e-health initiatives.
Sanders *et al.*[41]	UK	Back pain	A qualitative interview study of the perceptions of general practitioners towards the use of a new system for treating back pain.
May *et al.*[30]	UK	Telecare for chronic disease management in the community	Large-scale comparative study employing qualitative data collection techniques, including semi-structured interviews.
May *et al.*[31]	UK	Development of a simplified approach and web-enabled toolkit	A description of processes by which the authors developed a simplified approach of NPT for use by clinicians, managers, and policy makers, and which could be embedded in a web-enabled toolkit and online users manual.
Furler *et al.*[42]	Australia	Diabetes	A qualitative interview study exploring the use of insulin in general practice with a focus on barriers and enablers for timely initiation.
Bouamrane *et al.*[32]	UK	Remote and telehealth services	The authors outline a theoretical model of processes of intervention within the health services, and describe issues with the continued sustainability of existing models of care – and the potential opportunities for new technologies in addressing these challenges.
Spangaro *et al.*[43]	Australia	Screening for intimate partner violence (IPV) in Australian antenatal, mental health, and substance abuse services	Explores providers’ perceptions about the relevance of IPV to their role, the extent to which screening is routine, the existing challenges, the impact on clinical work or patient care, and the suggested changes to the policy.
Kennedy *et al.*[44]	UK	Delivering the WISE (Whole Systems Informing Self-Management Engagement) training package in primary care	Learning from formative evaluation, the purpose being to ensure that the WISE training package was robust and likely to be effective enough to be tested in an RCT.
Gunn *et al.*[45]	Australia	Embedding effective depression care: using theory for primary care organizational and systems change	Authors used a method informed by the principles of participatory action research (PAR) and utilized a mix of quantitative and qualitative methods to gather data about routine depression care in a range of primary care settings via: audit of electronic health records; observation of routine clinical care; and structured, facilitated whole-of-organization meetings.
Gask *et al.*[46]	UK	Collaborative care for depression?	Qualitative data collected in both focus groups and one-to-one interviews before and after an exploratory RCT of a collaborative model of care for depression.
Murray *et al.*[33]	UK	E-health	Reports on the development and formative evaluation of an e-Health Implementation Toolkit (e-HIT), which aims to summarize and synthesize new and existing research on implementation of e-health initiatives.
Wilkes and Rubin [47]	UK	Infertility management and primary care	A process evaluation of open access hysterosalpingography (HSG) utilizing the results of two qualitative studies (a focus group study and an in-depth interview study) and two quantitative studies (a pilot survey and a pragmatic cluster RCT).
Gask *et al.*[48]	UK	Mental health in primary care	A longitudinal qualitative multiple case study approach in a purposive sample of 12 organizations, chosen to reflect a maximum variety of organizational contexts for mental health care provision.
Elwyn *et al.*[49]	UK	Decision support technologies (DST)	A conceptual analysis of the outcomes of previous primary research and reviews to highlight implementation problems for DSTs in routine settings. Using a virtual working environment to examine: the ‘workability’ of DSTs in professional-patient interactions; how DSTs affect knowledge relations between their users; how DSTs impact on users' skills and performance; and the impact of DSTs on the allocation of organizational resources.
Mair *et al.*[50]	UK	Utilization of telecare in chronic lung disease	A process evaluation of a RCT of home telecare for the management of acute exacerbations of chronic obstructive pulmonary disease (COPD).
Morriss [51]	UK	Clinical guidelines for bipolar disorder	To critically review the evidence concerning the implementation of clinical guidelines for bipolar disorder.
May *et al.*[52]	UK	Process evaluation for complex interventions in primary care	A retrospective analysis of the implementation of two different complex trials: (i) the delivery of problem-solving therapies for psychosocial distress, and (ii) the delivery of nurse-led clinics for heart failure treatment in primary care.

### How is NPT being operationalized?

NPT, and its four related constructs, were utilized in over one-third of the papers reviewed (n = 11) [15,19,28,30-32,34,35,37,44],[45]. One paper [41] focused solely on the construct of Coherence. The remaining papers (n = 17) [5,18,29,33,36,38-40,42,43],[46-52] focused solely on the construct of Collective Action, or NPM as it was earlier known (see Table 2, column 2).

**Table 2:** The operationalization of NPT across the papers included in the review

**Author**	**Level of use model/theory**	**Application of NPM/NPT**	**Study participants in empirical papers**
Mair *et al.*[28]	NPT	As the literature under study focused on implementation processes rather than outcomes, the authors analyzed the extracted data qualitatively using NPT as a coding framework.	N/A
Blakeman *et al.*[34]	NPT	NPT provided a framework for generation and analysis of the data.	GPs and practice nurses
Franx *et al.*[35]	NPT	Related findings to NPT constructs.	Professionals (clinicians, healthcare staff including manager and team co-ordinator).
Ehrlich *et al.*[36]	Although NPT was the overarching theoretical framework used for the broader series of studies in this project, NPM was used specifically to aid data interpretation and the discussion in this study.	Interpretive analysis of interview data was conducted using NPT to structure data analysis and interpretation.	Professionals (nurses)
Finch *et al.*[15]	NPT	A 30-item instrument (Technology Adoption Readiness Scale (TARS)) for measuring normalization processes in the context of e-health service interventions was developed on the basis of NPT.	Professionals (First phase authors of published reviews of e-health; second phase nurses, call handlers, health info advisors, nurse advisors and others).
Gallacher *et al.*[19]	NPT	A secondary analysis of qualitative interview data, using framework analysis, informed by NPT.	Patient
Watson *et al.*[37]	NPT	All papers were coded using a framework analysis which evaluated the data in two ways using the 10 transition categories and four elements of Normalization Process Theory that are important for successful implementation and integration of healthcare interventions.	N/A
Forster *et al.*[5]	NPM	Survey and interview questions specific to the project were designed to reflect the four constructs of NPM in the implementation of the new model of care.	Professionals and patients (midwives and women)
Atkins *et al.*[38]	NPM	Data were analyzed initially using qualitative content analysis. The resulting categories were then organized under the constructs of the NPM.	Professionals and lay workers
Godden and King [29]	NPM	Analysis was supported by NPM. The principles of NPM were used to explore how successful implementation of proposed new technologies could be achieved.	Professionals (GPs, consultants, nurses, and others involved in respiratory care)
James [39]	NPT (Collective Action with an emphasis on Relational Integration and Interaction Workability related dimensions)	Created coding categories that were then examined under headings according to the NPM.	Practitioners and parents
MacFarlane and O’Reilly-de Brún [40]	NPM	The authors describe their actual use of NPM to inform research questions, sampling, coding and data analysis.	Professionals and patients (GPs and patients)
Murray *et al.*[18]	NPT collective action and its four subcomponents	Data were analyzed using the framework method according to four components of the Collective Action construct of NPT.	Professionals (staff with responsibility for planning and/or executing an e-health initiative—‘implementers’ were defined as any person charged with assisting with the implementation of an e-health system.
Sanders *et al.*[41]	NPT specific focus on coherence	Semi-structured interviews were organized around the four dimensions of the NPT: The analysis of the second stage interviews identified seven emergent themes, which were mapped onto the ‘Coherence’ construct within the NPT.	Professionals (GPs)
May *et al.*[30]	NPT	Framework analysis of qualitative data informed by NPT.	Professionals and patients (health professionals, managers, patient, carers, social care professionals and managers, and service suppliers and manufacturers)
May *et al.*[31]	NPT	Presented NPT to potential and actual users for review.	Professionals
Furler *et al.*[42]	NPM	Data analysis drew on the NPM in developing initial coding categories.	Professionals and patients (GPs, nurse educators and patients)
Bouamrane *et al.*[32]	NPT	Review of NPT and use in three e-health supporting case studies.	Professionals and patients (case study one: nurses, doctors, patient advocates, administrators, technologists, researchers)
Spangaro *et al.*[43]	NPT collective action and its four constructs	NPT was applied to the findings.	Professionals (staff and management)
Kennedy *et al.*[44]	NPT	NPT provided a framework for development of the intervention. NPT was used to give a focus to discussions and analysis, and reading of the interviews was undertaken in the context of the training observations and from the perspective of NPT.	Professionals (GPs, nurses, practice managers, clerical and reception staff)
Gunn *et al.*[45]	NPT	NPT identified as an analytical theory to guide the conceptual framework for implementing best practice depression care. Transcripts coded using interpretive framework of NPT.	Professionals (healthcare professionals, including receptionists, practice nurses, dieticians, nurse educators, psychologists and social workers)
Gask *et al.*[46]	NPM	The authors describe their actual use of NPM to inform research questions, coding, data analysis and interpretation.	Professionals and patients
Murray *et al.*[33]	NPM	The content of the e-HIT was derived by combining a theoretical framework with a literature review and new empirical data.	E-health experts and implementers
Wilkes and Rubin [47]	NPM	The results of two qualitative studies and two quantitative studies are interpreted by mapping the results to the NPM.	Professionals and patients
Gask *et al.*[48]	NPM (SSW and CI)	Framework analysis based on NPM. To examine the extent to which clinical governance of mental health care has been normalized within NHS primary care.	Professional – lay informant (clinical governance leads, mangers, audit leads and mental health leads; chief executive, and a lay informant)
Elwyn *et al.*[49]	NPM	NPM was used as the basis of conceptual analysis to examine the ‘workability’ of decision support technologies in professional-patient interactions. The authors sought to develop and refine the NPM through a concept analysis approach.	Physicians, patients and managers
Mair *et al.*[50]	NPM	A framework approach to data analysis was used.	Professionals (nurses) and patients
Morriss [51]	NPM	NPM was applied to analyze the NICE guideline recommendations for bipolar disorder.	N/A
May *et al.*[52]	NPM	Applied the NPM retrospectively to analyze trials of complex interventions in mental health and heart disease.	Professionals and patients

Our analysis of the application of the constructs across settings indicates that authors attributed meanings to each construct that, in general, resonated with our understanding of the constructs and had veracity in terms of their reported analysis and interpretation of data from their specific study setting.

For Coherence, there was a clear emphasis on understanding and conceptualization of interventions and their work (n = 8) [15,19,32,34,35,37,41,45];

For Cognitive Participation, the emphasis was on notions of legitimation and buy-in, both in terms of the individuals involved and involving others (n = 8) [15,19,28,32,34,35,37,45];

For Collective Action, the emphasis was on organizational resources, training and divisions of labor, confidence and expertise as well as the workability of the intervention in clinical interactions (n = 25) [5,15,18,19,28,29,32-40,42],[43,45-52];

For Reflexive Monitoring, the emphasis across studies was on appraising and monitoring implementation work (n = 9) [15,19,28,32,34,36,44,48],[52].

There were, however, exceptions to this general finding, which typically related to the overlap between constructs. For example, in Gunn *et al.*’s [45] paper we considered that data about the doubts practice nurses and receptionists had regarding their role in delivering depression care could fit with Cognitive Participation’s subcomponent legitimation*,* and could also fit with Collective Action’s subcomponent skill set workability*,* which relates to the division of labor and the allocation of tasks. Sanders *et al.*[41] also reported all their data about a new system for treating back pain under Coherence, although for us much of the data appeared to relate to Collective Action (because the data were based on data generated with study participants based on their experiences of doing implementation work). However, the main point is that irrespective of whether the issue fitted best in one construct or another, the framework helped to raise important issues.

In almost all cases NPT was used as an organizing framework for analyses and reporting findings. It was also used to inform study/intervention design [5,40,41,44,46], to generate research questions for fieldwork [39,41,45], and to create tools for investigating and supporting implementation (TARS and eHIT) [15,31] (see Table 2, column 3).

As presented within Table 2, column 4, while almost half of the studies (n = 13) [5,30,32,38-40,42,46-50,52] were multi-perspectival with involvement of professionals and service users, 12 of the remaining studies [15,18,29,31,33-36,41,43-45] focused on the perspectives of healthcare professionals only. However, it is evident that within the latter studies, different perspectives were often sought from within the healthcare profession (*i.e.*, GPs, nurses, allied health professionals, senior management).

It is interesting to note that Gallacher *et al.*’s study [19] is the only one to focus solely on the patient’s perspective. While not multi-perspectival, it does reinforce the message that the implementation of complex health interventions owes as much to the work of patients as it does to service providers and other personnel in health and social care agencies [31].

### What are the reported benefits, if any, of using NPT?

The majority of papers reviewed [5,15,18,19,28,30,34,35],[37-40,43,45-47,49-52] provided data about their experiences of using NPT, and the various challenges and benefits in using it.

MacFarlane and O’Reilly-de Brún [40] reflected on the challenge, for example, of overcoming tensions around using a predetermined conceptual framework and not wishing to ‘force data into predetermined codes or categories.’ They also described their concerns about understanding the constructs and ‘getting it right,’ stating that ‘it was sometimes difficult to know exactly what CI was about in this specific setting, and how it differed from SSW, and so on.’ The concern was that if the authors misunderstood the intended conceptual meaning of the constructs, their analysis would not be congruent with the NPM, and this would reduce the benefits of using the theory in the first place. Atkins *et al.*[38] and Franx *et al.*[35] shared similar views in that the application of the NPT constructs was problematic due to the aforementioned overlap and difficulty of discerning the differences between the constructs. Gunn *et al.*[45] remarked on the efforts required in developing each of the constructs within the complexity of current organizational practice.

Some authors commented on data falling outside of the NPT/NPM coding frame [19,28,50]. Mair *et al.*[28] noted that:

‘only 6% of issues fell outside of their coding framework, either because they were strictly technical and attitudinal or because they were so generic and vague, with accompanying contextual data, that it was not possible to determine whether the concept really lay outside the model or was simply too general to be coded.’

Gallacher *et al.*[19] commented on the fact that while very few data fell outside the NPT coding frame, those that did consisted of emotional work. Mair *et al.*[50] reflected on ‘attitudes’ and comment that this particular theme did not map onto the constructs of NPM. However, they acknowledged that unlike other theories such as the Theory of Planned Behavior, NPM relates to the work being done in interactions rather than focusing on how attitudes or intentions will affect work.

Overall, there was strong endorsement from researchers for the theory across a range of disciplines. Of the 20 papers that provided data about their experiences of using NPT, 15 [5,18,19,28,35,37-39,43,46],[47,49-52] commented that NPT was beneficial because it provided an explanatory theoretical framework for helping to identify factors that promote and inhibit implementation of complex interventions:

‘Our findings suggest that NPT provides a useful framework for understanding the processes that affect the implementation, embedding, and integration of new technologies into healthcare systems’ [18].

‘The findings suggest that NPT is a theoretical framework that facilitates understanding of experiences of health care work at the individual, as well as the organizational, level’ [19].

It was also emphasized that NPT had assisted in making clear recommendations for future implementation (n = 11) [5,18,28,35,38,39,45-47,49],[51]:

‘The model also assists in making clear recommendations for future implementation. This was important as this program was a pilot with a view to inform service decisions on whether and how to scale up the program across the province. We anticipated that the model could provide insights regarding the factors that would lead to normalization of the program’ [38].

Finally, some authors discussed the positive impact of NPT on trial design and development of an intervention (n = 3) [5,46,52], for example:

‘The NPM provides a useful structure for both guiding and analysing the process by which an intervention is optimized for testing in a larger scale trial or for subsequent wide-scale implementation’ [46].

At the same time, authors suggest further development of NPT [15,18,38,50] relating to: the determination of its value to guide the development of interventions for use in routine healthcare [18]; the need for study-specific measure/application of NPT [15]; the development of tools and methods to assist in the use of the NPM [38]; its potential to be used as a tool to assess the likelihood of future normalization of a complex intervention [50].

## Discussion

Across the 29 NPT papers included in this review, most of which were from the UK, there is evidence of a growing interest in the application of the NPT beyond its original field of e-health and telehealth. It is mainly being used to qualitatively analyze the implementation of complex interventions in a diverse range of healthcare settings.

Our analysis of the application of the NPT constructs across settings indicates that, overall, authors attributed meanings to each construct that resonated with our understanding of the construct and that had veracity in terms of their reported data from their specific study setting. We did have some queries about authors’ coding decisions; these related to potential overlaps between constructs, and also the issue of what stage in the implementation journey data related to. Indeed, some authors reported challenges of this nature when discussing their use of NPT. They described the difficulties experienced in assigning data, which can often be so closely interrelated, to a single category within the theory [38].

While it is valuable to note that the NPT constructs are not in competition with each other but are intended to work together to explain causal mechanisms, we do recognize the challenge of such coding decisions, which is inherent in all qualitative analyses [53]. Our own experience of NPT coding is that if data are based on planning the implementation of an intervention they are most likely to relate to Coherence and Cognitive Participation, and if they are based on actual experiences of enacting a new intervention they are most likely to relate to Collective Action and Reflexive Monitoring. At the same time, NPT has been developed with attention to the dynamic nature of implementation work (*e.g.*, sense making may be influenced by enactment). Therefore, we also acknowledge that the fluidity and flexibility inherent in NPT is important to take into account during coding, based on the specific context of each piece of data.

Overall, there was strong endorsement from several authors that it was beneficial to use NPT as a conceptual framework to analyze implementation processes and inform recommendations to guide implementation work [5,40]. This is an extremely important finding, suggesting that NPT is a new theory that does provide a generalizable framework that can be applied across contexts, with opportunities for incremental knowledge gain over time and an explicit framework for analysis that can explain and potentially shape implementation journeys. This finding about the benefit of using NPT is similar to Helfrich *et al.*’s [7] finding about the benefit of using the ‘Promoting Action on Research Implementation in Health Services’ framework, and thus we can see an expanding evidence base about the use of theory in studies about translational gaps.

A number of authors reported that some of their findings were outside the NPT conceptual framework. This is not a problem *per se* because the NPT, like any middle-range theory, cannot and does not claim to be a theory of everything. This finding indicates that authors are thinking critically about the relevance of NPT constructs to their data and are using it as a heuristic device rather than as a ‘conceptual straitjacket.’ Such critical and flexible use of NPT is recommended by its developers [31] and advocates of using theory in social science research more generally [54].

In terms of recommendations for future use of NPT, we note that authors rarely explained why they had selected NPT as opposed to other theories and rarely contrasted findings to previous studies. This may in part be a function of the current development of the implementation science literature, and the natural evolution of standards and expectations about what details researchers most need to report [7]. However, for future use it would be valuable to have this detail in the write-up of NPT or any theory. We therefore recommend that authors explicate their rationale for choosing NPT as their theoretical framework, particularly given that implementation science, like other closely related fields (*e.g.*, health services research, health technology assessment, and improvement science), needs comprehensive, robust, and rigorous theories that explain the social processes that lead from inception to practice [55]. In detailing their use of theory, authors will be making a contribution to implementation theory.

Also, even though NPT has highlighted the need to provide a whole-system analysis, many papers in this review only included single-stakeholder perspectives and there was an emphasis on service providers rather than service users. The limitations of such an approach to inform implementation processes should be considered during the analysis process [31] and acknowledged by researchers as they develop recommendations for future research or practice.

Finally, to fully explore the scope of NPT to shape implementation journeys, we need more studies that use the theory in a prospective manner. In this review there was only one such study [38], but others are underway and will provide valuable findings in the future [6]. As suggested by Grol *et al.*[56] and Murray *et al.*[10], there is scope for NPT to be used during the planning stages of implementation projects to explore the real-world context in which the work will take place. Such approaches may provide important data to re-direct or stop planning if the likelihood of normalization is low.

### Limitations

First, this review did not include non-English language papers and therefore we cannot comment on the use and perceived value of NPT in non-English speaking settings. However, it would be valuable to do so in future reviews and to explore its use and stability across cultural settings, given that our search identified a number of foreign language papers using NPT from countries such as Sweden and Italy and the use of NPT in three European-wide research projects: REsearch into implementation STrategies to support patients of different ORigins (RESTORE) [6], Self-care Support for People with Long Term Conditions, Diabetes and Heart Disease: A Whole System Approach (EU-Wise) [57], and INnovative, Midlife INtervention for Dementia Deterrence (In-MINDD) [58].

Second, reviews are one step removed from the primary data, and therefore we rely on the authors’ reports of benefits and limitations of NPT usage, which could be limited or sanitized versions of their experience. No attempts were made to contact authors for additional information.

In terms of gaps in the information provided by the studies, we noted that in the quality appraisal process the lowest scoring domain tended to be in terms of ethics and bias, primarily because there was very limited discussion of either of these issues within the papers. As an example, while authors reported whether they had ethical approval or not, they did not elaborate on ethical issues in the research process. This limited reporting about ethics and bias is likely to be a function of the word count restrictions in journal articles.

To enhance quality and rigor, we took several steps to increase the transparency and reliability of our review. First, given that some of the authors (LB, COD, FSM and AMacF) have been involved in the development of NPT, we decided at the outset of the review that we would focus on explicit accounts of NPT in use and explicit reflections by authors on its merits/demerits (*i.e.*, to allow the authors of the NPT papers to speak for themselves as much as possible). We favored this approach on the basis that it would heighten the authenticity of our conclusions given our involvement in the development of NPT. Second, all steps of the review were led by the lead author who has had no prior involvement in the development of the theory. Third, during the coding process we employed double independent coding during indexing and carting of the data, and discussions with all authors to reach consensus during the mapping and interpretation phases. These three steps heightened our critical thinking during the analysis process and the authenticity of our conclusions.

As this was a qualitative review of predominantly qualitative empirical studies, some aspects of the PRISMA [59] statement were not applicable (see Additional file 4).

## Conclusions

In conclusion, NPT has served as a useful and beneficial conceptual heuristic for many researchers and practitioners from different communities in terms of framing and enhancing analysis of implementation processes and informing recommendations for improving implementation.

NPT has potential to help understand the translational gap, providing us with a generalizable framework that can apply across differing settings and individuals, the opportunity for incremental accumulation of knowledge, and an explicit framework for analysis. Whether NPT can serve as a tool to shape implementation processes in ways that will promote integration and embedding of complex interventions remains unclear and merits investigation.

## Abbreviations

CI: Contextual integration; CKD: Chronic kidney disease; COPD: Chronic obstructive pulmonary disease; DST: Decision support technologies; HSG: Hysterosalpingography; In-MINDD: INnovative, midlife INtervention for dementia deterrence; IPV: Intimate partner violence; IW: Interactional workability; NPM: Normalization process model; NPT: Normalization process theory; PAR: Participatory action research; RESTORE: REsearch into implementation STrategies to support patients of different ORigins; RCT: Randomized controlled trial; RI: Relational integration; SSW: Skill set workability; TARS: Technology adoption readiness scale.

## Competing interests

LB, FM, AMacF, and COD have been part of an international group of academics who have contributed to the development of NPT. LB is a member of the Advisory Board of Implementation Science. Editorial decisions regarding publication of this manuscript were made independently by another editor.

## Authors’ contributions

RM and AMacF were responsible for overall coordination; RM, LB and AMacF conceived of the study; all authors (RM, AMacF, LB, SM, FSM, COD) participated in the study design, coding of data, analysis and drafting of the manuscript; while RM, AMacF and LB undertook the independent quality appraisal process. All authors read and approved the final manuscript.

## Supplementary Material

Additional file 1 **PICO Table.** A qualitative systematic review of studies using the Normalization Process Theory. Due to the qualitative nature of the literature being reviewed, PICO as a search strategy did not neatly fit with our research questions so we adapted the PICO Table to include the following criteria: participants, study design and collection approach, interventions, analysis, aims/discussion and outcomes. Click here for file

Additional file 2 **Electronic Search.** Presents the literature review’s interface engine, search screen, databases, and the overall search strategy. Click here for file

Additional file 3 **Quality Appraisal Template and Result.** Drawing on Hawker, Payne, *et al.*[27], each of the 29 papers was subjected to a quality appraisal process undertaken by three of the authors. This file presents both the template used and the result for each of the papers appraised. Click here for file

Additional file 4 **Completed PRISMA Statement.** As this was a qualitative review of predominantly qualitative empirical studies, some aspects of the PRISMA [59] statement were not applicable. Click here for file
