# Supplementary material for: A qualitative systematic review of studies using the normalization process theory to research implementation processes
Source: Implement Sci. 2014 Jan 2;9:2. doi: 10.1186/1748-5908-9-2 (PMC3905960; doi:10.1186/1748-5908-9-2)
Supplement: Additional file 1 — PICO Table. A qualitative systematic review of studies using the Normalization Process Theory. Due to the qualitative nature of the literature being reviewed, PICO as a search strategy did not neatly fit with our research questions so we adapted the PICO Table to include the following criteria: participants, study design and collection approach, interventions, analysis, aims/discussion and outcomes. [file 1748-5908-9-2-S1.pdf]

PICO Table: A Qualitative Systematic Review of the Literature on Normalization Process Theory in use

Author	Title	Participants	Study Design & Collection Approach	Interventions	Analysis	Aim/Discussion	Outcomes
Atkins, S. Lewin, S. Ringsberg K. and Thorson A. [38]	Provider experiences of the implementation of a new tuberculosis treatment programme: A qualitative study using the normalisation process model.	All nurses (n=6) and adherence counsellors (n=6) working on the programme were asked to participate in interviews. All treatment supporters (approximately 85) were invited through adherence counsellors to participate in focus group discussions (FGDs).	A qualitative approach was used. Interviews and focus group discussions.	Using the NPM as an analytic framework, this study aims to explore staff perceptions of a new TB treatment programme modelled on the ART treatment programme.	Data were analysed initially using qualitative content analysis. The resulting categories were then organised under the constructs of the NPM.	Aims to explore staff perceptions of a new TB treatment programme modelled on the ART treatment programme.	The NPM assisted in categorising the challenges experienced during implementation of the TB Treatment Programme. The results suggest that issues remain that need to be resolved before the programme is implemented more widely. Considerable work is needed in order to embed the TB Treatment Programme in routine clinic practice.
Blakeman, T., Protheroe, J., Chew-Graham, C., Rogers, A. and Kennedy, A. [34]	Understanding the management of early stage chronic kidney disease in primary care.	In total, 21 out of the 28 health professionals (75%) invited agreed to participate. The final sample was spread across 11 practices and comprised 11 GPs (six male, five female; median age 45 years [range 30–62 years]) and 10 nurses (all female; median age 47.5 years [range 39–60 years]). Eighteen out of the 21 participants were directly involved in the CLAHRC CKD collaborative	A qualitative study using semi structured interviews.	Using NPT as a framework, the study constructs an understanding of how early-stage CKD is encountered and dealt with in general practice.	NPT was used to sensitise the analysis to the work being carried out in primary care. Initial coding of the data was undertaken independently by three of the authors and categories were identified by comparing these codes. Using the coding framework to help operationalise the theory, these constructs were applied to the sets of practices outlined above, concerning the management of CKD in primary care. The coding framework assisted comparative analysis of data from each individual account, matched interviews, and across the dataset out in primary care.	To explore processes underpinning the implementation of CKD management in primary care.	The study builds on previous findings concerning the disclosure of CKD in primary care. This study sought to understand the work undertaken by GPs and practice nurses in implementing a relatively new phenomenon into general practice. The findings highlight tensions experienced by professionals surrounding the management of individuals with early-stage CKD. These tensions need to be considered when developing interventions to improve the delivery of care for people with mild and moderately ‘low kidney function’, vascular conditions, or multimorbidity. . NPT provided a framework to explore in greater detail the interactional work that occurs in clinical encounters and within an organisation.

Bouamrane, M, Osbourne, J., Mair, F.S. [32]	Understanding the implementation and integration of remote and telehealth services…an overview of NPT.	**Case study 1**: 68 interviews with: consultant dermatologists (19), tele-dermatology nurses (11), doctors (3), patients advocates (4), administrators (8), technologists (6) and researchers (17) (these figures refer to the number of interviews as there were multiple interviews with a single practitioner in certain cases). **Case study 2**: 12 specialist respiratory nurses and 104 patients took part in the RCT trial. 9 patients and 11 nurses**. Case study 3**:15 professionals (healthcare, technical & managers) & 22 patients.	Review of NPT and supporting case studies. **Case study 1**: Qualitative approach using interviews; **Case study 2**: Qualitative approach using interviews; **Case study 3:** Qualitative approach using interviews.	**Review of NPT and supporting case studies: Case study 1**: Finch, Mair & May analysed 12 tele-dermatology studies over 8 years (1997-2005); **Case study 2**: a qualitative study embedded in a RCT of a remote monitoring system for people with acute chronic obstructive pulmonary disease (COPD); **Case study 3**: Qualitative study of a telepsychiatry system.	The authors review the application of NPT to three selected case studies; teledermatology, tele-monitoring of patient with chronic lung disease a tele-psychiatry.	The authors begin by describing issues with the continued sustainability of existing models of care – and the potential opportunities for new technologies in addressing these challenges. This is followed in by a description of the NPM and the theory which subsequently developed from this model: the NPT. The authors review the application of NPT to 3 selected case-studies: tele-dermatology, tele-monitoring of patient with chronic lung disease a tele-psychiatry. And conclude with a discussion and directions for future development of the theory.	Case studies that have normalisation of e-health services were successful when certain facilitating factors as per NPT were in place.
Ehrlich, C. Kendall, E. , Winsome S.J.	How does care coordination provided by registered nurses ‘‘fit’’ within the organisational processes and professional relationships in the general practice context?	Eleven general practices, who were members of a single division of general practice in South-East Queensland, Australia, were invited to participate. Six general practices met the selection criteria (that is, an experienced RN was involved, general practice managers supported the intervention, general practice management systems were organised and maintained, RNs had access to necessary resources, and the general practice could demonstrate capacity to meet the demands of the project). One general practice withdrew prior to commencement of the intervention. A total of nine RNs from five general practices participated in the study. Six participants were general practice RNs (one general practice was represented by two RNs), and three were RN GPLOs who provided pivotal support to general practice RNs.	A qualitative interpretive research design, which used focus group interviews with nurses using a semi-structured interview protocol.	To explore how registered nurse (RN)-provided care coordination could move beyond implementation to become embedded and integrated within the organisational processes and professional relationships of the general practice context.	Interpretive analysis of interview data was conducted using NPT, particularly NPM, to structure data analysis and interpretation.	The aim of this study was to develop understanding about how a registered nurse-provided care coordination model can ‘‘fit’’ within organisational processes and professional relationships in general practice.	Within teams of health care providers, interventions need to be workable and able to be integrated if they are to ‘fit’ within the context and become part of routine practice. Interventions such as RN-provided care coordination, which was piloted in this study, are more likely to become part of routine practice if they: (a) confer an interactional advantage, (b) equal or improve relational integration through accountability and confidence within networks, (c) improve skill-set workability by calibrating to an agreed skill-set at a recognizable location in the division of labour, and (d) support contextual integration by conferring an advantage on an organisation in flexibly executing and realizing work (May, 2006).

Elwyn, G., Legare, F. et. al. [49]	Ardous implementation: Does the Normalisation Process Model explain why it's so difficult to embed decision support technologies for patients in routine clinical practice?	Physicians, patients and managers.	The NPM was used as the basis of conceptual analysis of the outcomes of previous primary research and reviews. Using a virtual working environment the authors applied the model and its main concepts to examine: the 'workability' of DSTs in professional-patient interactions; how DSTs affect knowledge relations between their users; how DSTs impact on users' skills and performance; and the impact of DSTs on the allocation of organizational resources.	Through a conceptual analysis of the outcomes of previous primary research and reviews, authors aimed to decide whether the NPM was of value in understanding the difficulties encountered in getting DSTs embedded into practice.	NPM was used as the basis of conceptual analysis	To test the conceptual adequacy of the model in understanding the difficulties encountered in getting DSTs embedded into practice.	One of the main insights gained by applying the NPM was the need to consider its propositions from the perspective of different actors, particularly when DSTs is an inherent component of interactions between the actors.
Finch, T., Mair, F., O Donnell, C., Murray, E. and May, C. [23]	From theory to 'measurement' in complex interventions: Methodological lessons from the development of an e-health normalisation instrument	**Phase 1:** A total of 63 participants completed the expert survey out of 252 invitations (24% response) that were presumed to be received (subtracting invitations returned as ‘undeliverable’). **Research background**: Medical 32; Social science 24; Informatics 21; Nursing 11; Economics 2; Health Services Research 5; Non-specific 6. **Gender:** Male 59 and Female 41. **Location of residence**: USA 37; UK 27; Canada 13; Europe (excluding Scandinavia) 10; Australia/New Zealand 8; Scandinavia 6. **Phase 2: At Site 1,** 46/243 participants completed the survey (19% response rate). 100% female. **At Site 2,** 231/1351 (17% response rate) completed the survey sufficiently for inclusion in the analysis.86% female & 14% male.	The instrument was pre-tested in two health care settings in which e-health (electronic facilitation of healthcare decision-making and practice)was used by health care professionals.	A 30-item instrument (Technology Adoption Readiness Scale (TARS)) for measuring normalisation processes in the context of e-health service interventions was developed on the basis on Normalization Process Theory (NPT). NPT focuses on how new practices become routinely embedded within social contexts. T		This paper aimed to (1) describe the process and outcome of a project to develop a theory-based instrument for measuring implementation processes relating to e-health interventions; and (2) identify key issues and methodological challenges for advancing work in this field.	The practical output of this study was the development of the TARS instrument, which was intended to enable researchers and practitioners to quantify a range of processes proposed by the NPT to contribute to the successful normalisation of e-health, either as a ‘diagnostic’ tool or for evaluation purposes
Forster, D., Newton, M., McLachlan, H., Willis, K. [5]	Exploring implementation and sustainability of models of care: can theory help?	**Case study 1:** One thousand women were recruited to the team midwifery trial between February 1996 and November 1997. Eight midwives were recruited from volunteers among the existing midwifery staff in the hospital, and team midwifery care was provided following the same clinical	The authors use two case studies where new models of maternity care were implemented and evaluated via RCTs to discuss how)or whether) the use of theory might inform implementation and sustainability strategies	Two case studies provide the opportunity to reflect on the implementation of two midwifery models of care at the Royal Women’s Hospital (the Women’s), a tertiary hospital in	The authors demonstrate how the NPM was applied in planning of the evaluation phases of the RCT as a means of exploring the implementation of the caseload model of care. They argue that a theoretical	Authors discuss how the Normalisation Process Model was applied in planning of the evaluation phases of the RCT as a means of exploring the implementation of the caseload model of care. The authors argue that a theoretical understanding of issues related to implementation and sustainability can make a valuable contribution when researching	The Normalisation Process Model has provided a framework within the COSMOS trial to examine some of these issues prospectively, both through the evaluation research design (relating to the implementation of the model of care into practice) and analysis of findings. Organisations may use the evidence from the trial findings to guide implementation strategies, ensuring that constructs that have been identified as important for the

		protocols and guidelines as standard care. **Case study 2:** Recruitment of 2314 women to the trial took place from September 2007 to June 2010, with the last birth in December 2010 (primary outcomes reported elsewhere). Midwives already employed at the Women's were offered first preference to work in the caseload model, then external advertising was used to fill further vacancies (to a total of approximately 12 full time equivalent positions).		Melbourne, Australia, and consider how NPT can be used to understand the barriers and enablers to sustainability of new and complex models of care within maternity care settings.	understanding of issues related to implementation and sustainability can make a valuable contribution when researching complex interventions in complex settings such as hospitals.	complex interventions in complex settings such as hospitals.	model's sustainability are encompassed in implementation strategies. In addition, future trials of models of care may benefit from using the NPT not only to understand implementation, but to guide trial design and development of the intervention.
Franx, G. Oud, M., de Lange, J. Wensing, M. and Grol, R. [35]	Implementing a stepped-care approach in primary care: results of a qualitative study	Participants for the study were selected from thirteen multidisciplinary primary care teams participating in the depression Quality Improvement Collaborative (QIC). These thirteen QIC teams had been recruited throughout the country by a national QIC project team on the basis of the following criteria: the team had a multidisciplinary structure, there was sufficient motivation and time for all members to participate, and a local team coordinator was available. Although team members sometimes had worked together in another context, most of them had not worked together as a depression team prior to the QIC. At the start of the QIC, all teams were asked to participate in the intervention study and the process evaluation, alongside their implementation work. Five teams did not wish to spend extra time on research activities and declined. Eight teams consented, consisting of PCPs, primary care psychologists, social workers, mental health nurses, physiotherapists, consulting psychiatrists and psychotherapists, local	An intervention study using a controlled before and after design was performed. Part of the study was a process evaluation utilizing a semi structured group interviews, to provide insight into the perceptions of the participating clinicians on the implementation of stepped care for depression into their daily routines.	Since 2004, 'stepped-care models' have been adopted in several international evidence-based clinical guidelines to guide clinicians in the organisation of depression care. To enhance the adoption of this new treatment approach, a Quality Improvement Collaborative (QIC) was initiated in the Netherlands. Alongside the QIC, an intervention study using a controlled before-and-after design was performed. Part of the study was a process evaluation, utilizing semi-structured group interviews, to provide insight into the perceptions of the participating clinicians on the implementation of stepped care for depression into their daily routines.	Analysis was supported by NPT, with reference to Gunns NPT framework on depression.	The qualitative process analysis presented here aims to add to the quantitative findings as it documents the way in which the intervention was received and implemented by clinicians, and identifies the factors associated with reception and implementation.	By relating the findings to the NPT constructs, the authors were able to provide another layer to the findings. The constructs provide the authors with sensitizing concepts that could lead to a better understanding of the findings of this process evaluation, as well as guide additional recommendations on how to conduct implementation projects in depression care.

		managers, and team coordinator.					
Furler, J., Spitzer, O., Young, D. and Best, J. [42]	Insulin in general practice Barriers and enablers for timely initiation	Ten general practitioners, four diabetes nurse educators and 12 patients were interviewed.	A qualitative study using semi structured, in-depth interviews.	Insulin in general practice: Barriers and enablers for timely initiation	Data analysis drew on the NPM in developing initial coding categories.	This study explores barriers and enablers to insulin initiation in general practice.	Normalisation process theory provides a framework for analysis of the findings through focusing on how the 'work' of diabetes care is understood, given meaning, undertaken and supported.
Gallacher, K., May, C. et al. [18]	Understanding Patient's Experiences of Treatment Burden in Chronic Heart Failure Using Normalization Process Theory	Participants were 47 patients with chronic heart failure managed in primary care in the United Kingdom who had participated in an earlier qualitative study about living with this condition.	Secondary analysis of qualitative interview data.	To assess the burden associated with treatment among patients living with chronic heart failure and to determine whether NPT is a useful framework to help describe the components of treatment burden in these patients	Framework analysis, informed by NPT	To use NPT to identify, describe, and understand the components of treatment burden experienced by patients with chronic heart failure.	Suggests that NPT is a useful theoretical framework for understanding patients experiences of illness and health care services, and their active contribution to their overall care and self care.
Gask, L. Bower, P. Lovell, K. Escott, D., Archer, J., Gilbody, S. Lankshear, A. Simpson, A., Richards, D. [46]	What work has to be done to implement collaborative care for depression? Process evaluation of a trial utilizing the NPM.	A convenience sample of stakeholders was recruited from primary care organizations (PCOs) in the north of the UK. Primary Care Physicians (PCPs) and practice nurses were recruited from practices in PCOs that had agreed to participate in the trial. Other participants were recruited from teams and specialist care providers that provided primary and secondary mental healthcare to the PCOs. Patients were recruited by four participating PCPs who each mailed a letter to 20 of their patients who were receiving treatment for depression in primary care. No participants had had any experience of this method of organizing care and none been involved in the trial design. The authors interviewed 49 participants. All 38 professionals who were asked to participate in the study agreed to do so: 12 PCPs, four psychiatrists, four clinical psychologists, four practice nurses and 14 mental health workers (seven mental health nurses, two	Application of the NPM to qualitative data collected in both focus groups and one to one interviews before and after an exploratory randomised controlled trial of a collaborative model of care for depression.	This study utilises the Normalisation Process Model (NPM) to inform the process of implementation of collaborative care in both a future full-scale trial, and the wider health economy.	Framework analysis, based on the NPM.	Aim to apply the NPM to the process data in order to consider what can be learnt about the additional or 'hidden' work (i.e., that which is not immediately apparent at conception of the project or not usually included in publication of results of a trial) that needs to be done to make a collaborative care intervention for depression in primary care both workable and integrated into routine practice in both our forthcoming full-scale trial of collaborative care for depression in the UK and the wider healthcare settings following the trial. In initiating this task, the authors were particularly interested in the value of application of the NPM to process data in order to aid in the further development and evaluation of this intervention in the UK.	The NPM provided a neat and conceptually rich framework to guide analysis and our thinking about a range of key issues in the implementation of collaborative care for depression in both research trials and routine practice. It provided a novel way of evaluating and interpreting process data that added value to the analysis. Using the model, it was possible to observe that certain predictions about work that would need to be done that could be made from analysis of the pre-trial data relating to the four different factors of the NPM were borne out in the post-trial data.

		counsellors, three graduate mental health workers, one social worker, and one unqualified support worker). Most interviews were conducted individually apart from two focus groups with 11 of the 14 mental health staff. From the 80 letters posted to patients, 17 consented to participate of which 11 were interviewed, five subsequently declined or could not be contacted, and one became so distressed that the interview was abandoned on ethical grounds and the patient was encouraged to contact the PCP.					
Gask, L. Rogers, A. et al. [48]	Beyond the limits of clinical governance? The case of mental health in English primary care.	Interviews with clinical governance leads and managers (12 interviews carried out with 17 informants), audit leads (3 interviews) and mental health leads (11 interviews with 18 informants- one site could not identify a lead) to explore how implementation of clinical governance had progressed. Additionally the authors interviewed informants identified as by PCT informants as 'primary care' leads at the local Mental Health provider trust. For three of the sites, as the Trust was an integrated provider of primary care and mental health, there was no need to conduct a further interview. A further site arranged a single group interview with four representatives from both primary care and mental health. Four interim interviews with mental health leads in both PCTs and mental health trusts were carried out at two sites during 2001, selected on the basis of the case study profiles because they might provide contrasting views of the development of primary	A longitudinal qualitative multiple case-study approach in a purpose sample of 12 PCTs	To explore the quality of primary care for people with mental health problems through the new institutional processes of 'clinical governance'.	Framework analysis, based on the NPM.	To examine the extent to which clinical governance of mental health care has been normalised within NHS primary care.	The NPM predicts that in order to become normalised, new working practices such as clinical governance activities have to satisfy four (sets of) conditions. The contested nature and status of 'mental health' within primary medical care makes it particularly difficult to change clinical working practices and the ways in which patients and professionals themselves interact, i.e. to satisfy the interactional workability and relational integration, insofar as they apply to clinical governance activities. It also compounds the (more substantial) skill-set and contextual problems and uncertainties faced by those who seek to 'improve the quality of primary mental health care'. The data show a lack of clear conceptualisation about what primary mental health care is or ought to be, under defined roles and wide professional discretion, especially for GPs. They also suggest that clinical governance and the mental health NSF only weakly satisfy the NPM's contextual integration conditions. This is not for want of willingness on senior managers' or clinicians' parts but more due to lack of knowledge about what (material and human) resources are required and how they can be used to integrate clinical governance activity, including NSF implementation, more centrally into mental health care; and how to start bridging the service gaps noted above.

		these sites were unchanged from the previous year, however by 2003–4 the PCT mental health leads had changed in all but 3 of the 11 sites for which they could identify a lead person. A total of 41 interviews carried out with 49 informants form the main empirical material for the analysis.					
Godden, D. and King, G. [29]	Rational development of telehealth to support primary care respiratory medicine: patient distribution and organisational factors.	Semi-structured interviews (n=20) were conducted with health professionals and managers likely to be involved in implementation of telehealth. These key informants – including GPs, consultants, nurses, and others involved in respiratory care – had responsibilities across the region (including urban, rural and remote areas) and they were recruited purposively.	Qualitative interviews with health professionals (n=20) focussing on the potential for telehealth in respiratory medicine were analysed using the NPM.	The potential for telehealth in respiratory medicine.	Analysis was supported by NPM	The study examined the potential for applying telehealth in a region of the UK by exploring the distribution of patients and examining attitudes to implementation of telehealth.	The main perceived barriers to implementation in the study were in skill set workability – mainly training issues – and contextual integration – mainly about costs. Interestingly, realisation – an element of contextual integration which refers to moving responsibilities between professional groups – was not a prominent concern, perhaps reflecting the extent to which this is already happening in rural communities as, for example, nurses and paramedics take on roles previously delivered by doctors. However there is evidence that actual implementation of telecare services can face major problems of interactional workability where nurses lack confidence in the technological approach adopted.
Gunn, J. Palmer, V. et al. [45]	Embedding effective depression care: using theory for primary care organisational and systems change.	Seven eligible primary care organisations were identified. Each had from two up to ten or more GPs working within them plus other professionals (receptionists, practice nurses, dieticians, diabetic nurse educators, psychologists, and social workers). Five organisations were privately owned by principal GPs, one was a corporate owned health centre, and one was a publicly funded community health centre.	Authors used a mixed method, observational approach to gather data about routine depression care in a range of primary care settings via: audit of electronic health records; observation of routine clinical care; and structured, facilitated whole of organisation meetings.	To identify the components of an effective model of depression care.	NPT identified as an analytical theory to guide the conceptual framework development	To identify the components of an effective model of depression care. Work is presented as a conceptual framework to guide how to implement organisational and systems change in mental health care reform in primary care.	Identifies NPT as suitable for the task of providing an analytical theory to develop a conceptual framework to guide the implementation of an effective model and system of depression care.

James, D.M. [39]	The Applicability of Normalisation Process Theory to Speech and Language Therapy: A review of qualitative research on a speech and language intervention.	There were five papers that presented data on the parents' or therapists' views. Three of these papers used semi-structured interviews or focus groups to elicit participants' views on the intervention. Two of the papers presented data from questionnaires that were used to elicit parental views of speech and language therapy. These studies were included because they explored parental views on direct (traditional) versus indirect (such as the HPP) approaches with children and families. **Girolametto, Tannock and Siegel** (1993); Mothers who had taken part in a HPP N = 32. **Glogowska and Campbell** (2000) Parents who had taken part in a RCT to evaluate traditional SLT intervention in pre-school children N = 16 selected respondents according to the logic of maximum variation. **Glogowska, Campbell, Peters, Roulstone and Enderby** (2001); Parents who had taken part in a RCT to evaluate traditional SLT intervention in pre-school children. N = 89. **Baxendale, Frankham and Hesketh** (2001); Parents who had taken part in a controlled study to compare HPP with traditional clinic-based SLT N = 37 in total**. Pennington and Thomson** (2007) SLTs who deliver the HPP in the UK N = 16.	Secondary analysis of qualitative interview data.	Speech and Language Therapy	Framework analysis, informed by NPT	The primary aim of this study was to test the applicability of the propositions on the role of collaborative work laid out in the NPM and NPT to the context of speech and language therapy so that, if found to be applicable, the NPT could be used to inform the design of new intervention research in the field.	The analysis based on the NPM uncovered interpersonal processes between the practitioners and parents that were likely to have given rise to successful implementation of the intervention. In previous qualitative research on this intervention where the Medical Research Council's guidance on developing a design for a complex intervention had been used as a framework, the interpersonal work within the intervention had emerged as a barrier to implementation of the intervention. It is suggested that the design of services for children and families needs to extend beyond the consideration of benefits and barriers to embrace the social processes that appear to afford success in embedding innovation in healthcare.

Kennedy, A. Chew-Graham, C. et al. [44]	Delivering the WISE Training Package in Primary Care: Learning from formative evaluation.	Practices with more than two GPs were identified within a Primary Care Trust (PCT). The practices who agreed to take part in the study were asked to select two training dates where all staff (GPs, nurses, practice managers, and clerical and reception staff) could be present for a three-hour training session.	Observation, audio recordings, and face to face semi structured interviews were conducted.	Using the NPM as an analytic framework, this study aims to learn from the formative evalution of the Whole System Informing Self-management Engagement (WISE) approach which encompasses creating, finding, and implementing appropriate self-care support for people with long-term conditions.	Framework analysis informed by NPT.	Aimed to refine the patient, practitioner, and patient level components of the WISE approach and translate the principles of WISE into an operational intervention deliverable through NHS training methods	The formative evaluation approach and attention to normalisation process theory allowed the training team to make adjustments to content and delivery and ensure appropriate staff attended each session.
MacFarlane, A. and Oreilly De Brun, M. [40]	A reflexive account of using a theory-driven conceptual framework in qualitative health research.	18 General practitioners; 4 General practice administrators; 2 Independent interpreters; 2 Service user representatives; 14 Service user representatives; 1 Company manager. The emphasis for sampling and recruitment was on identifying information-rich cases, participants who were understood to have knowledge and experiences relevant to the phenomenon under investigation (Patton, 1990). The funders of the study had a particular interest in the experiences and views of general practitioners, and the original study remit included general practitioners as the sole participant group. However, the authors encouraged inclusion of a broader group of participants so that experiences and views of implementation and normalization could be examined across stakeholder groups. This was prompted by our experience of using a multiperspectival approach in qualitative research.	The authors present a reflective paper on their decisions about whether or not to use the NPM, and describe their actual use of it to inform research questions, sampling, coding, and data analysis.	A reflexive account of using a theory-driven conceptual framework in qualitative health research.	NPM was used as the basis of conceptual analysis.	The authors provide a reflexive account of Their experience of using a theory-driven conceptual framework, the NPM, in a qualitative evaluation of general practitioners' uptake of a free, pilot, language interpreting service in the Republic of Ireland.	The added value of the NPM analysis was that the authors understanding of individual themes became more insightful and advanced, and the interpretation of themes was enhanced. The NPM offered an organizing principle to "think with our data" in a very specific way; i.e., to think about the layered meanings of individual themes in relation to predescribed macro- and micro-level issues, and the mediating relations between these, and thus to be more alert to the complex processes of implementation and conditions for normalization. In a sense, it provided an element of the researcher analyst role in that it offered an outline of analytic and theoretical categories to advance the descriptive analysis (Gibbs, 2007).

Mair, F., Hiscock, J. and Beaton, S. [50]	Understanding factors that inhibit or promote the utilization of telecare in chronic lung disease.	Patients (n=9) and specialist respiratory nurses (n=11) providing the telecare service in chronic lung disease.	Participant observation of activities involved in the implementation and delivery of the home telecare service. Semi-structured interviews were carried out with patients (*n* = 9) and nurses (*n* = 11) participating in a RCT.	A process evaluation of a randomized controlled trial (RCT) of home telecare for the management of acute exacerbations of chronic obstructive pulmonary disease (COPD), using the NPM as an explanatory framework.	A framework approach to data analysis was used.	The telecare service did not provide an interactional advantage for the nurses providing this service and did not fit with the nurses' views of the most appropriate or preferred use of their skills. The telecare service seemed unlikely to become normalized as part of routine healthcare delivery, because the nursing team lacked confidence that it was a safe way to provide healthcare in this context and it was not perceived as improving efficiency. The telecare intervention failed to satisfy three of the four constructs of the NPM, namely interactional workability, relational integration, and skill-set workability.	The NPM effectively mapped onto the study findings and explained those factors that inhibited the routine delivery of COPD services by telecare.
Mair, F., May, C., O'Donnell, C., Finch, T., Sullivan, F. & Murray, E. [28]	A systematic review of reviews of e-health implementation studies, focusing on implementation processes rather than outcomes.	From 8206 unique citations screened, the authors excluded 7973 on the basis of the title or abstract and retrieved 233 full-text articles. Of these, 37 met the inclusion criteria. Of note, 20 of these reviews were published between 1995 and 2007 and 17 were published in the following two years.	An explanatory systematic review.	A systematic review of reviews of e-health implementation studies, focusing on implementation processes rather than outcomes.	The authors have interpreted the results in the light of an explanatory framework –NPT, that specifies mechanisms of importance in implementation processes.	This review not only collates and summarizes data but also analyses it and interprets it within a theoretical framework, NPT. The authors approach has allowed them to explore the factors that facilitate and hinder implementation, identify gaps in the literature and highlight directions for future research. In particular, this work highlights a continued focus on organizational issues, which, despite their importance, are only one among a range of factors that need to be considered when implementing e-health systems.	Content analysis of the 37 reviews identified 801 attributive statements about implementation processes that could be interpreted using NPT as an explanatory framework.
May, C. Finch, T. et. al. [30]	Integrating telecare for chronic disease management in the community: What needs to be done?	Drawn from telecare services in community and domestic settings in England and Scotland, 221 participants were included, consisting of health professionals and managers (n=22); patients and carers (n=31); social care professionals and managers (n=90); and service suppliers (n=11) and manufacturers (n=67).	Large scale comparative study employing qualitative data collection techniques: semi-structured interviews with key informants, task-groups, and workshops.	To identify factors inhibiting the implementation and integration of telecare systems for chronic disease management in the community.	Framework analysis informed by NPT	To identify the policy and practice factors that affect the routine incorporation of telecare into everyday practice, and to explore the ways that these factors promoted or inhibited the implementation and integration of telecare systems.	The study revealed the ways that multiple cycles of uncertainty run through implementation processes, and inhibit the embedding and integration of new ways of delivering care. The authors present a model of the analysed data that shows how uncertainties were derived from problems of coherence and participation as per NPT. This follows the key storylines that ran through participants accounts. Along each storyline are a series of nodes that identifies a factor that inhibits the normalization of telecare systems in practice and which appeared in respondents accounts.

May, C. Finch, T., Ballini, L., MacFarlane, A., Mair, F. Murray, E. Treweek, S. and Rapley, T. [32]	Evaluating complex interventions and health technologies using NPT: development of a simplified approach and web enabled tool kit.	Multiple potential users, researchers and practitioners, of NPT (n=60).	Presented NPT to potiential and actual users. (ii) created a simplified set of statement and explanations expressing core constructs of the theory (iii) circulated statements of a criterion sample of 60 researchers, clinicians and others to collect qualitative data about their criticisms of the statements (iv) reconstructed statements and explanations to meet users criticisms.	Development of a simplified version of NPT for use by clinicians, managers, and policy makers, and which could be embedded in a web-enabled toolkit and on-line users manual.	The authors treated the comments about the value and limits of the NPT toolkit as attributive statements and analysed them using a simple and descriptive thematic analysis.	The paper describes the processes by which the authors developed a simplified version of NPT for use by clinicians, managers and policy makers and which could be embedded in a web enabled toolkit and online users manual.	Provides a user friendly version of NPT that can be embedded in a web enabled toolkit and used as a heuristic device to think through implementation and integration problems.
May, C. Mair, F. Dowrick, C. and Finch, T. [52]	Process evaluation for complex interventions in primary care: Understanding trials using the NPM.	Not specified.	NPM is applied to two complex trials: the delivery of problem solving therapies for psychosocial distress and the delivery of nurse-led clinics for health failure treatment in primary care.	Case studies of randomized controlled trials of complex interventions in primary care form useful opportunities to explore at a *general* level how the NPM might be applied. In this study the authors draw on two such examples: the delivery of problem solving therapies for psychosocial distress and the delivery of nurse-led clinics for health failure treatment in primary care.	Explores its application to two bodies of research around the effectiveness and implementation of complex interventions – problem solving therapies for people with depression and nurse-led heart failure clinics in primary care.	The Normalization Process Model (NPM) is an evaluation model that asks what people *do* to make a complex intervention workable, and to integrate it in practice. The paper develops this by first discussing the development of the theoretical model, and then applies it to two case studies of complex trials that combine both treatment and organizational interventions in primary care.	Application of the NPM shows how process evaluations need to focus on more than the immediate contexts in which trial outcomes are generated. Problems relating to intervention workability and integration also need to be understood. NPM may be used effectively to explain the implementation process in trials of complex interventions.
Morriss, R. [51]	Implementing clinical guidelines for bipolar disorder	Not applicable	A Medline search was made of the literature on the implementation of guidelines in bipolar disorder and mental health. The implementation of nonpharmacological treatments from the 2006 NICE Guideline for Bipolar Disorder was then used as a case example.	To critically review the evidence concerning the implementation of clinical guidelines for bipolar disorder.	NPM of complex interventions was applied to the NICE guideline recommendations for bipolar disorder.	To critically review the evidence concerning the implementation of clinical guidelines for bipolar disorder. May's normalization process model can be used to predict how easily a guideline recommendation will be to implement, so that it becomes embedded in routine clinical practice. Furthermore, it can also be used to identify the nature of the barrier to its implementation into practice.	Without a detailed local understanding of the barriers to implementation as per NPM and the resources and will to overcome them, there is likely to be a considerable local variation in the implementation of guidelines for bipolar disorder.

Murray, E,. May, C and Mair, F. [33]	Development and formative evaluation of the e-Health Implementation Toolkit (e-HIT).	In the first stage of the evaluation, those interviewed included senior clinicians, managers and academics each of whom had extensive experience of e-health implementations within the NHS (n = 13). In the second stage of the formative evaluation, the revised toolkit was circulated by e-mail to the implementers who had been interviewed in the qualitative study (n = 23).	A two stage formative evaluation of the e-HIT was undertaken. For the first stage, the prototype e-HIT was circulated to a group of e-Health experts. These experts were asked to complete the e-HIT for an e-Health initiative they had personal experience of, and on the basis of this experience, to comment critically on the e-HIT. In the second stage of the formative evaluation, the revised toolkit was circulated by e-mail to the implementers who had been interviewed in the qualitative study (n = 23). Participants were asked to comment on the likely usefulness of the e-HIT, make suggestions for improvement, and whether it adequately reflected their own experience.	The development and formative evaluation of an e-Health Implementation Toolkit (e-HIT) which aims to summarise and synthesise new and existing research on implementation of e-Health initiatives, and present it to senior managers in a user-friendly format.	Data derived from interviews were summarised, synthesised and combined with the constructs from the NPM.	The aim of the e-HIT was to summarise and synthesise research evidence on factors that impede or facilitate implementation of e-Health initiatives and present this evidence in a format that could be easily digested and used by staff considering or planning an e-Health implementation. The aim of this paper is to describe the process of development and formative evaluation of e-HIT, and describe the final toolkit, in line with recent calls for more detailed descriptions of the processes and content of complex interventions.	The e-HIT shows potential as a tool for enhancing future e-Health implementations. Further work is needed to make it fully web-enabled, and to determine its predictive potential for future implementations.

Murray, E. Burns, J. et al. [17]	Why is it difficult to implement e-health initiatives? A qualitative study	The authors purposively recruited a maximum variety sample, aiming to include senior Department of Health or Connecting for Health staff with responsibility for a number of e-health projects across multiple organizations, senior staff from within the Trust or Health Board with lead responsibility for implementing a number of e-health systems within their organization (such as chief executive officers), and middle management with day-to-day responsibility for the implementation under study. Recruitment within each case study continued until the authors reached saturation, (i.e., until no new data were emerging from subsequent interviews. Based on previous experience, the authors estimated that up to ten interviews per case study would be needed. Twenty-three interviews were undertaken: ten for Case Study 1, five for Case Study 2, and eight for Case Study 3.	Qualitative data collection: semi structured interviews. Three case studies selected to provide a range of healthcare contexts.	To explore and understand the experiences of implementers and their assessment of factors which promote or inhibit the successful implementation, embedding, and integration of e-health initiatives, as per the constructs outlined in NPT.	Data were analyzed using the framework method proposed by Ritchie and Spencer, according to four components of the collective action construct of NPT (May 2006): Data were coded to the four constructs and overall degree of normalization.	To explore and understand the experience of implementers and their assessment of factors that promote or inhibit successful implementation, embedding and integration of e-health initiatives.	NPT with its emphasis on collective action provided a good explanation for the observed variability in normalisation of three contrasting technologies in different contexts.
Sanders, T., Foster, N., Bie Nio Ong [41]	Perceptions of general practitioners towards the use of a new system for treating back pain: a qualitative interview study	General practitioners (GPs) before (n = 32) and after (n = 9) the introduction of a subgrouping for targeted treatment system.	A qualitative interview survey. The GP interviews were embedded within a prospective, population-based, quality improvement study comprising three phases: (a) assessment of GPs' and physiotherapists' attitudes and behaviours regarding low back pain, (b) a quality improvement intervention comprising educational courses, regular feedback sessions, and the installation of computerised and paper-based systems for subgrouping for targeted treatment system, and (c) assessment of GPs' and physiotherapists' attitudes and behaviours regarding low back pain and the	The interviews were nested within a larger study (the IMPaCT Back ('IMplementation study to improve PAtient Care through Targeted treatment for Back pain') study), which aimed to evaluate the improvement in quality of care for back pain patients following implementation of a subgrouping for targeted treatment system	Data analysed using constant comparison drawing upon insights and development connections between themes. Adopted the NPT to explain the uptake of the new system and to examine the relevance of coherence for the implementation of innovations in organisations.	The key obstacle to implementation of the new subgrouping for targeted treatment system for low back pain in primary care was an initial failure to achieve 'coherence' of the desired practice change with GPs. Despite this, GPs used the tool to different degrees, though this signified a general commitment to participating in the study rather than a deeper attitude change towards the new system.	The task of integrating new ways of working in healthcare settings can be challenging [28]. According to the NPT the implementation of a new approach is operationalised through four mechanisms: The GPs in this study did not progress beyond the first stage of implementation, or coherence, the main focus of this paper. In summary, low back pain was generally perceived as an 'uninteresting' and clinically unchallenging health problem by GPs, which may partly explain their lack of engagement with it. In working from NPT, the adoption of a new way of working by GPs was partly determined by the meaning that they ascribed to it (i.e. Coherence), and any perceived change to the stability and continuity of routine medical work could be met with resistance. Therefore, an appreciation of such routines is the first step towards

			subgrouping for targeted treatment system.				understanding the perceived acceptance of innovations. The second is a familiarity with how a new way of working may affect work patterns; and, the third is the impact that it may have on interpersonal relationships with peers. Failure to adequately understand all three dimensions may result in largely unsuccessful attempts at integrating new ways of working in the NHS.
Spangaro, J., Poulos, R., and Zwi, A. [43]	Pandora Doesn't Live Here Anymore: Normalization of Screening for Intimate Partner Violence in Australian Antenatal, Mental Health, and Substance Abuse Services	Ten focus groups were conducted with 59 participating health workers: 5 were conducted in antenatal clinics (29 participants); 4 were held at substance abuse services (23 participants); and 1 was conducted at a mental health service (7 participants). Six male and 53 female participants had been screening for an average of 4.4 years. Despite the policy requiring staff training, only 48 participants (81%) had been trained. The remainder had commenced in their roles subsequent to the instigation of the policy and had not been included in repeat training.	A qualitative study using focus groups.	This study used focus groups with health care providers undertaking screening in an established program to understand challenges, and enablers of screening apply this to a model of how health policies become routinized in practice.	The analysis employed an inductive approach involving immersion in the data to derive patterns and interrelationships, confirmation of relationships, and synthesis of key findings (Patton, 2002). *Normalization process theory*, developed to explain how complex health interventions become embedded in practice, was also applied to the findings.	Normalization of Screening for Intimate Partner Violence in Australian Antenatal, Mental Health, and Substance Abuse Services.	Both the sustained screening rate reported by these services and the responses of the workers in this study point to a policy that has become the normalized practice. The authors suggest that it is not solely the static presence of these four elements described by May (2006) that has led to the normalization of this policy, but an interactional effect, over time. In their application of May's schema, it appears that training and referral pathways were initially important and remain so for maintenance. However, it appears that "normalization" was most strongly brought about through *familiarity* acting as a mechanism from the use of brief, scripted, visible questions, further reinforced by women's favorable reactions.
Watson, R, Parr, J., Joyce, C., May, C. and A.S. Le Couteur [37]	Models of transitional care for young people with complex health needs: a scoping review	The database searches and expert recommendations yielded 350 papers. Using the study inclusion and exclusion criteria, 19 papers (reporting 18 service models) met the study criteria. These included 14 diabetes service models from Australia, USA, Canada, UK, Italy, Spain and Denmark; four service models for young people with CP from the USA and UK. All of the CP models included young people with a range of physical disabilities and CHNs (including CP). The excluded papers included reviews of services provision rather than	Scoping review: using search terms concerning transitional care, four databases were systematically searched for papers published in English between 1980 and April 2010. Additional informal search methods included recommendations from colleagues working with young people with each of the three conditions and making contact with clinical and research teams with expertise in transitional care. Inclusion and exclusion criteria were applied to define the papers selected for review. A separate review of policy documents, adolescent health	To identify successful models of transitional care for young people with CHNs. Three conditions were used as exemplars: cerebral palsy, autism spectrum disorders and diabetes.	All papers were coded using a framework analysis which evaluated the data in two ways using the 10 transition categories and four elements of Normalization Process Theory that are important for successful implementation and integration of healthcare interventions.	To identify models of transitional care from child to adult health services for CP, ASD and diabetes, using a broad range of literature including peer reviewed publications (1980–April 2010); To seek evidence to inform 'best practice' about transitional care for children with CHNs; To investigate whether the identified models of transitional care have been evaluated; To use Normalization Process Theory to evaluate whether aspects of service sustainability had been considered.	The scoping review identified no models of transitional care for young people with ASDs, either from electronic database searches or from UK professionals working in the field. Services lacked evaluation, with only a minority reporting consideration of sustainability of the service. NPT provided a structure to assess key elements required for successful implementation and integration of new practice into everyday healthcare.

		descriptions of specific services, papers focussing on other conditions and service recommendations. No models of transitional care for young people with ASD were identified from database searches or expert recommendations.	and transition literature was also undertaken; 10 common summary categories for the components of high quality services were identified. Nineteen papers were selected for review.				
Wilkes, S. and Rubin, G. [47]	Process evaluation of infertility management in primary care: has open access HSG been normalised?	**Four empirical studies: (i) Pilot Study**: Using hospital clinical records, the authors tracked the outcome of all infertile couples from the six pilot practices over a nine-month period. (ii) **Focus group study**: The authors purposively selected three focus groups to provide a range of GPs' views. In total, 13 practitioners participated: 11 GPs, one GP registrar and one Nurse practitioner. (iii) **Pragmatic cluster-randomized controlled trial:** 670 infertile couples presented to 33 intervention practices and 25 control practices. **In-depth interview study:** 39 interviewees: 12 GPs, 5 fertility specialists and 13 infertile couples (9 interviewed with their partner).	The results of two qualitative studies and two quantitative studies evaluating open access HSG are interpreted by mapping the results to the NPM.	Process evaluation of infertility management in primary care.	The results of four empirical studies evaluating open access HSG in the initial management of infertility in general practice were mapped to the NPM constructs.	To map the results of four empirical quantitative studies to the NPM to explain why open access hysterosalpingography (HSG) for the initial management of infertile couples has or has not normalized in primary care	The NPM has given an explanation why open access HSG has been adopted but not normalized into everyday general practice. The challenge, now, is to define and address the constructs within the model that are limiting normalization and propose methods to address these barriers. Modeling a complex intervention and mapping the barriers is currently a process seldom undertaken in complex intervention trials (Bosch et al., 2007). The NPM has been a useful tool to describe the likely normalization of open access HSG for the initial management of infertility in primary care..
