# Supplementary material for: A qualitative systematic review of studies using the normalization process theory to research implementation processes
Source: Implement Sci. 2014 Jan 2;9:2. doi: 10.1186/1748-5908-9-2 (PMC3905960; doi:10.1186/1748-5908-9-2)
Supplement: Additional file 2 — Electronic Search. Presents the literature review’s interface engine, search screen, databases, and the overall search strategy. [file 1748-5908-9-2-S2.pdf]

Interface - EBSCOhost

Search Screen - Advanced Search

Database - Academic Search Complete;AMED - The Allied and Complementary Medicine Database; Biomedical Reference Collection: Expanded; CINAHL Plus with Full Text; MEDLINE; OmniFile Full Text Mega (H.W. Wilson);PsycARTICLES; PsycINFO; Social Sciences Full Text (H.W. Wilson);UK & Ireland Reference Centre

	Query	Limiters/Expanders	Last Run Via	Results
S2	"Normalization Process Theory" OR "Normalization Process Model"	Limiters - Scholarly (Peer Reviewed) Journals<br>Search modes - Boolean/Phrase	Interface - EBSCOhost<br>Search Screen - Advanced Search<br>Database - Academic Search Complete;AMED - The Allied and Complementary Medicine Database;Biomedical Reference Collection: Expanded;CINAHL Plus with Full Text;MEDLINE;OmniFile Full Text Mega (H.W. Wilson);PsycARTICLES;PsycINFO;Social Sciences Full Text (H.W. Wilson);UK & Ireland Reference Centre	28
S1	"Normalization Process Theory" OR "Normalization Process Model"	Search modes - Boolean/Phrase	Interface - EBSCOhost<br>Search Screen - Advanced Search<br>Database - Academic Search Complete;AMED - The Allied and Complementary Medicine Database;Biomedical Reference Collection: Expanded;CINAHL Plus with Full Text;MEDLINE;OmniFile Full Text Mega (H.W. Wilson);PsycARTICLES;PsycINFO;Social Sciences Full Text (H.W. Wilson);UK & Ireland Reference Centre	46
