# Supplementary material for: A qualitative systematic review of studies using the normalization process theory to research implementation processes
Source: Implement Sci. 2014 Jan 2;9:2. doi: 10.1186/1748-5908-9-2 (PMC3905960; doi:10.1186/1748-5908-9-2)
Supplement: Additional file 3 — Quality Appraisal Template and Result. Drawing on Hawker, Payne, et al.[27], each of the 29 papers was subjected to a quality appraisal process undertaken by three of the authors. This file presents both the template used and the result for each of the papers appraised. [file 1748-5908-9-2-S3.pdf]

This checklist is from Hawker, S., S. Payne, et al. (2002). "Appraising the Evidence: Reviewing Disparate Data Systematically." Qualitative Health Research 12(9): 1284-1299.

Please assess each paper on the following criteria. For scoring please refer to notes below.

Good=4
Fair=3
Poor=2
Very poor=1
Lower scores =poor quality

Notes for appraising the quality of each paper:

1. Abstract and title:
Did they provide a clear description of the study?
Good Structured abstract with full information and clear title.
Fair Abstract with most of the information.
Poor Inadequate abstract.
Very Poor No abstract.

2. Introduction and aims:
Was there a good background and clear statement of the aims of the research?
Good Full but concise background to discussion/study containing up-to date literature review and highlighting gaps in knowledge. Clear statement of aim AND objectives including research questions.
Fair Some background and literature review. Research questions outlined.
Poor Some background but no aim/objectives/questions, OR Aims/objectives but inadequate background.
Very Poor No mention of aims/objectives. No background or literature review.

3. Method and data:
Is the method appropriate and clearly explained?
Good Method is appropriate and described clearly (e.g., questionnaires included). Clear details of the data collection and recording.
Fair Method appropriate, description could be better. Data described.
Poor Questionable whether method is appropriate. Method described inadequately. Little description of data.
Very Poor No mention of method, AND/OR Method inappropriate, AND/OR No details of data.

4. **Sampling:**
Was the sampling strategy appropriate to address the aims?
Good Details (age/gender/race/context) of who was studied and how they were recruited. Why this group was targeted. The sample size was justified for the study. Response rates shown and explained.
Fair Sample size justified. Most information given, but some missing.
Poor Sampling mentioned but few descriptive details.
Very Poor No details of sample.

5. **Data analysis:**
Was the description of the data analysis sufficiently rigorous?
Good Clear description of how analysis was done. Qualitative studies: Description of how themes derived/ respondent validation or triangulation. Quantitative studies: Reasons for tests selected hypothesis driven/ numbers add up/statistical significance discussed.
Fair Qualitative: Descriptive discussion of analysis. Quantitative.
Poor Minimal details about analysis.
Very Poor No discussion of analysis.

6. **Ethics and bias:**
Have ethical issues been addressed, and what has necessary ethical approval gained? Has the relationship between researchers and participants been adequately considered?

Good Ethics: Where necessary issues of confidentiality, sensitivity, and consent were addressed. Bias: Researcher was reflexive and/or aware of own bias.
Fair Lip service was paid to above (i.e., these issues were acknowledged).
Poor Brief mention of issues.
Very Poor No mention of issues.

7. Results:
Is there a clear statement of the findings?
Good Findings explicit, easy to understand, and in logical progression. Tables, if present, are explained in text. Results relate directly to aims. Sufficient data are presented to support findings.
Fair Findings mentioned but more explanation could be given. Data presented relate directly to results.
Poor Findings presented haphazardly, not explained, and do not progress logically from results.
Very Poor Findings not mentioned or do not relate to aims.

8. **Transferability or generalizability:**
Are the findings of this study transferable (generalizable) to a wider population?
Good Context and setting of the study is described sufficiently to allow comparison with other contexts and settings, plus high score in Question 4 (sampling).
Fair Some context and setting described, but more needed to replicate or compare the study with others, PLUS fair score or higher in Question 4.Poor Minimal description of context/setting.
Very Poor No description of context/setting.

9. **Implications and usefulness: How important are these findings to policy and practice?**
Good Contributes something new and/or different in terms of understanding/insight or perspective. Suggests ideas for further research. Suggests implications for policy and/or practice.
Fair Two of the above (state what is missing in comments).
Poor Only one of the above.
Very Poor None of the above.

Author	Title	Journal	Year	Quality Approved Total score allocation Maximum score = 36
Atkins, S. Lewin, S. Ringsberg K. and Thorson A.	Provider experiences of the implementation of a new tuberculosis treatment programme: A qualitative study using the normalisation process model	BMC Health Services Research	2011	31
Bouamrane, M, Osbourne, J., Mair, F.S.	Understanding the implementation and integration of remote and telehealth services…an overview of NPT	Pervasive Computing Technologies for Healthcare	2011	31
Elwyn, G., Legare, F. et. al.	Ardous implementation: Does the Normalisation Process Model explain why it's so difficult to embed decision support technologies for patients in routine clinical practice?	Implementation Science	2008	25
Forster, D., Newton, M., McLachlan, H., Willis, K.	Exploring implementation and sustainability of models of care: can theory help?	BMC Public Health	2011	19
Franx, G. Oud, M., de Lange, J. Wensing, M. and Grol, R.	Implementing a stepped-care approach in primary care: results of a qualitative study	Implementation Science	2012	33
Gallacher, K., May, C. et al.	Understanding Patient's Experiences of Treatment Burden in Chronic Heart Failure Using Normalization Process Theory	Annals of Family Medicine	2011	34
Gask, L. Bower, P. Lovell, K. Escott, D., Archer, J., Gilbody, S. Lankshear, A. Simpson, A., Richards, D.	What work has to be done to implement collaborative care for depression? Process evaluation of a trial utilizing the NPM	Implementation Science	2010	29
Gask, L. Rogers, A. et al.	Beyond the limits of clinical governance? The case of mental health in English primary care.	BioMed Central	2008	25
Godden, D. and King, G.	Rational development of telehealth to support primary care respiratory medicine: patient distribution and organisational factors	Primary Care Respiratory Journal	2011	18
Gunn, J. Palmer, V. et al.	Embedding effective depression care: using theory for primary care organisational and systems change.	Implementation Science	2010	33
James, D.M.	The Applicability of Normalisation Process Theory to Speech and Language Therapy: A review of qualitative research on a speech and language intervention	Implementation Science	2011	23

Kennedy, A. Chew-Graham, C. et al.	Delivering the WISE Training Package in Primary Care: Learning from formative evaluation.	Implementation Science	2010	32
MacFarlane, A. and Oreilly De Brun, M.	A Reflexive Account of Using a Theory-Driven Conceptual Framework in Qualitative Health Research	Qualitative Health Research	2011	35
Mair, F., Hiscock, J. and Beaton, S.	Understanding factors that inhibit or promote the utilization of telecare in chronic lung disease	Chronic Illness	2008	30
May, C. Finch, T. et. al.	Integrating telecare for chronic disease management in the community: What needs to be done?	BMC Health Services Research	2011	34
May, C. Finch, T., Ballini, L., MacFarlane, A., Mair, F. Murray, E. Treweek, S. and Rapley, T.	Evaluating complex interventions and health technologies using NPT: development of a simplified approach and web enabled tool kit.	BMC Health Services Research	2011	35
May, C. Mair, F. Dowrick, C. and Finch, T.	Process evaluation for complex interventions in primary care: Understanding trials using the NPM.	BioMed Central	2007	31
Morriss, R.	Implementing clinical guidelines for bipolar disorder	Psychology and Psychotherapy: Theory, Research and Practice	2008	26
Murray, E,. May, C and Mair, F.	Development and formative evaluation of the e-Health Implementation Toolkit (e-HIT)	BMC Medical Informatics and Decision Making	2010	33
Murray, E. Burns, J. et al.	Why is it difficult to implement e-health initiatives? A qualitative study	Implementation Science	2011	33
Sanders, T., Foster, N., Bie Nio Ong	Perceptions of general practitioners towards the use of a new system for treating back pain: a qualitative interview study	BMC Medicine	2011	33
Watson, R, Parr, J., Joyce, C., May, C. and A.S. Le Couteur	Models of transitional care for young people with complex health needs: a scoping review	Child: care, health and development	2011	31
Wilkes, S. and Rubin, G.	Process evaluation of infertility management in primary care: has open access HSG been normalised?	Primary Health Care Research & Development	2009	27
Finch, T., Mair, O Donnell, Murray and May	From theory to 'measurement' in complex interventions: Methodological lessons from the development of an e-health normalisation instrument	BMC Medical Research Methodology	2012	34

Ehrlich, C. Kendall, E. , Winsome S.J.	How does care coordination provided by registered nurses ‘‘fit’’ within the organisational processes and professional relationships in the general practice context?	Collegian (2012)	2012	32
Furler, J., Spitzer, O., Young, D. and Best, J.	Insulin in general practice Barriers and enablers for timely initiation	Reprinted from Australian Family Physician	2012	33
Blakeman, T., Protheroe, J., Chew-Graham, C., Rogers, A. and Kennedy, A.	Understanding the management of early stage chronic kidney disease in primary care.	BMJ of General Practice	2012	33
Mair F, May C, O'Donnell C, Finch C, Sullivan F, Murray E:	Factors that promote or inhibit the implementation of e-health systems: an explanatory systematic review.	Bulletin of the World Health Organization	2012	32
Spangaro J, Poulos GR, Zwi BA:	Pandora doesn’t live here anymore: normalization of screening for intimate partner violence in Australian antenatal, mental health, and substance abuse services.	Violence and Victims	2011	31
