# Supplementary material for: A qualitative systematic review of studies using the normalization process theory to research implementation processes
Source: Implement Sci. 2014 Jan 2;9:2. doi: 10.1186/1748-5908-9-2 (PMC3905960; doi:10.1186/1748-5908-9-2)
Supplement: Additional file 4 — Completed PRISMA Statement. As this was a qualitative review of predominantly qualitative empirical studies, some aspects of the PRISMA [59] statement were not applicable. [file 1748-5908-9-2-S4.pdf]

Additional File 4: Completed PRISMA statement re. A Qualitative Systematic Review of the Literature on Normalization Process Theory in use

As this was a qualitative review of predominantly qualitative empirical studies, some aspects of the PRISMA statement [1] were not applicable.

Section/Topic	#	Description	Page No.
Title	1	A systematic qualitative review of the literature on Normalization Process Theory in use.	1
**ABSTRACT**			
Structured summary	2		3
**INTRODUCTION**			
Rationale	3		5-6
Objectives	4		Additional file 2: PICO Table
**METHODS**			
Protocal & registration	5	No Protocol registered	N/A
Eligibility criteria	6		8-9
Information sources	7		8
Search	8		8<br>Figure 2: PRISMA Flow Chart
Study selection	9		8
Data collection process	10		9
Data items	11		N/A
Risk of bias in individual studies	12		8
Summary measures	13		N/A
Synthesis of results	14		N/A
Risk of bias across studies	15		N/A
Additional analyses	16		N/A
**RESULTS**			
Study selection	17	Flow diagram developed (see Figure 2)	8<br>See Figure 2
Study	18		See Tables 1 & 2

characteristics			
Risk of bias within studies	19		8
Results of individual studies	20		Additional File 2 PICO Table
Synthesis of results	21		N/A
Risk of bias across studies	22		N/A
Additional analysis	23		N/A
**DISCUSSION**			
Summary of evidence	24		Tables 2 & 3
Limitations	25		17
Conclusions	26		17-18
FUNDING			
Funding	27		20

1. Liberati A, Altman D, Tetzlaff J, Mulrow C, Gøtzsche P, Ioannidis J, Clarke M, Devereaux P, Kleijnen J, Moher D: **The PRISMA statement for reporting systematic reviews and meta-analyses of studies that evaluate health care interventions: explanation and elaboration**. *BMJ* 2009, **339: b2700**.
